# The synergistic bactericidal effect of vancomycin on UTMD treated biofilm involves damage to bacterial cells and enhancement of metabolic activities

**DOI:** 10.1038/s41598-017-18496-3

**Published:** 2018-01-09

**Authors:** Jian Hu, Ning Zhang, Lifang Li, Ning Zhang, Yanfen Ma, Chedong Zhao, Qian Wu, Ying Li, Nianan He, Xiaoqin Wang

**Affiliations:** 1 Department of Clinical Laboratory, First Affiliated Hospital of Xi’an Jiaotong University, 277# West Yanta Road, Xi’an, 710061 Shaanxi province P.R. China; 2 Department of Emergency, First Affiliated Hospital of Xi’an Jiaotong University, 277# West Yanta Road, Xi’an, 710061 Shaanxi province P.R. China; 3 Department of Clinical Laboratory, Shaanxi Kang Fu Hospital, 52# Second Electronic Road, Xi’an, 710065 Shaanxi province P.R. China; 4 Department of Ultrasound, Anhui Provincial Hospital of Anhui Medical University, 4# Lujiang Road, Hefei, 230001 Anhui province P.R. China

## Abstract

In this study, the synergistic effect of vancomycin, a cell wall synthesis inhibitor, and ultrasound-targeted microbubble destruction (UTMD), on cell viability of *Staphylococcus epidermidis*, embedded in biofilm, was investigated. Biofilms are the leading causes of antibiotic-resistant bacterial infections of medical implants and prosthetics worldwide. The antibiotic-resistant nature of biofilm-embedded pathogens poses a critical challenge to the medical community. Previously, studies have demonstrated the efficacy of using ultrasound waves and UTMD in circumventing this problem. However, the mechanism(s) underlying this phenomenon was not clear. Here, the present study showed that both ultrasound and UTMD damaged the cell wall structure of *S*. *epidermidis*, and floccules and fragments from damaged cells were observed on transmission electron microscope micrograph. However, the cell membrane integrity was not seriously affected by treatments, and the treatment increased the metabolic activity levels of the dormant biofilm-embedded bacteria, detected by confocal laser scanning microscope and flow cytometry, which could make them susceptible to the effect of the antibiotic. Thus, the biological mechanism underlying the efficacy of the combined treatment involving UTMD and vancomycin in the case of *S*. *epidermidis* biofilm was dissected, which may be utilized for further investigations on other biofilm pathogens before clinical use.

## Introduction

In recent years, medical implant-related infections have emerged as challenging problems with the extensive use of medical implants and prosthetics (such as prosthetic valve, artificial catheter, intravascular stent, bone prosthesis, etc.) in clinical approaches. Implant-related infections are different from ordinary bacterial infections as even antibiotics that are effective against isolated pathogenic bacteria do not cure these infections^1^. Replacement of the indwelling medical devices after such infection is generally necessary. This has increased patient suffering and caused extensive economic wastage.

A large number of studies revealed that the leading cause for the emergence of antibiotic refractory bacteria is the formation of bacterial biofilms, which renders it increasingly resistant to multiple antibiotics and host defenses^1–5^. Biofilms are bacterial communities that adhere to biological or abiotic substrata, and are stabilized by extracellular polymeric substances, typically composed of polysaccharides, proteins, and extracellular DNA^1,3,6–8^. Compared to planktons, bacteria in biofilms differ in physiological state, metabolic activity, regulation of gene transcription, and tolerance to antibiotics, and significantly enhance the host’s immune system^3,5,9,10^. Therefore, biofilm-associated infections always cause refractory and persistent infections in clinic, and development of effective and non-invasive methods of treating biofilm-associated infections is urgently required.

Studies have shown that low intensity ultrasound of physiotherapy level can enhance the transfer efficiency of various drugs or biological macromolecules in tissues or cells without any damage to human tissues^11–13^. In addition, studies showed that low energy ultrasound has similar biological effects on bacteria, i.e., it improves the lethal effect of antibiotics on drug-resistant bacteria or biofilms^14–19^. Investigations regarding the effectiveness of antibacterial substances combined with ultrasonic therapy in the treatment of biofilm infection is now a research hotspot, and certain preliminary clinical studies have already been performed in dentology and surgery^20–25^. Other studies found that use of “microbubbles”, a common ultrasound contrast agent, as the cavitation nuclei can reduce the threshold of ultrasound cavitation and significantly enhance its biological effect compared to ultrasound treatment alone^26,27^. Ultrasound-targeted microbubble destruction (UTMD) obviously improved the uptake efficiency of macromolecules by eukaryotic cells^27–29^. Our previous study also showed that UTMD promoted the activity of a biofilm-resistant antibiotic to produce strong biofilm eradicating action^30^.

Recently, certain studies investigated the mechanism of the UTMD-assisted biofilm killing effect of antibiotics, and demonstrated that ultrasound or UTMD can destroy the biofilm matrix structure and promote drug delivery into the biofilm^31–34^. However, whether ultrasound or UTMD can directly affect the physiological state of bacteria in biofilm and reduce its drug resistance is still under investigation. In our current study, we used the biofilm of *Staphylococcus epidermidis* as a model, and explored the biological effect of ultrasound or UTMD on bacterial survival in biofilms to provide a theoretical basis for the use of ultrasound or UTMD in improving the efficacy of antibiotic treatment on implanted prosthetic-related biofilm infections.

## Results

### Basic characteristics of the *S*. *epidermidis* clinical isolate

The selected clinical strain of *S*. *epidermidis* used in this study was isolated from a central venous catheter of a patient admitted in our hospital. According to drug sensitivity analysis, this strain is resistant to multiple antibiotics but is sensitive to vancomycin (MIC = 1.0 μg/mL, detected by VITEK 2 compact system). Similar to *S*. *epidermidis* RP62A, this clinical strain forms a thick biofilm on polystyrene and glass surfaces, and the mature biofilm was resistant to 100 μg/mL vancomycin (100-fold MIC, 25 μg vancomycin per mg biofilm mass approximately).

### Effects of ultrasound and UTMD on the biofilm morphology of the *S*. *epidermidis* clinical isolate

**Figure 1:**
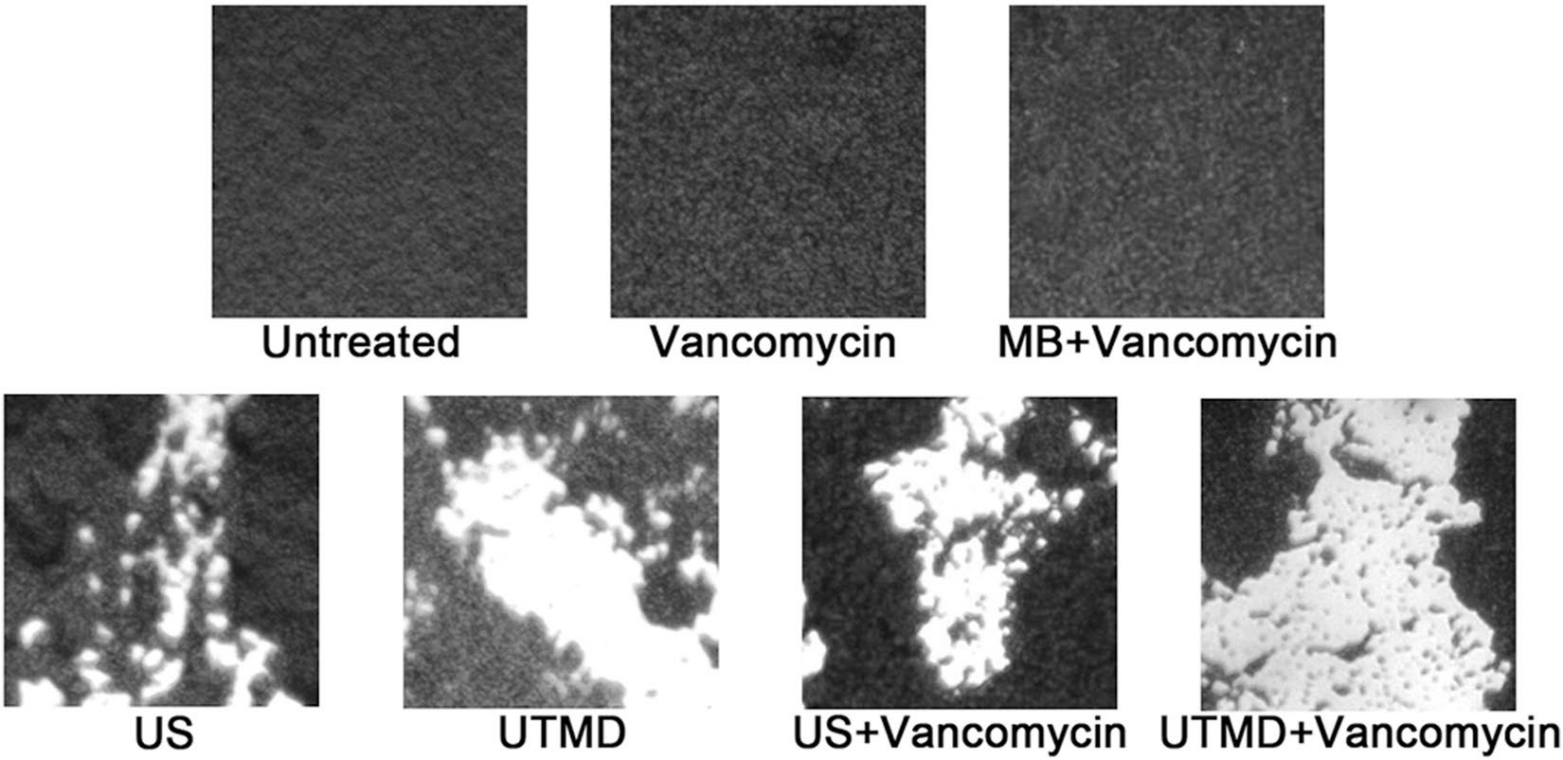
Optical morphology of crystal violet-stained biofilm of the *S*. *epidermidis* clinical isolate (20× objective). MB indicates microbubble treatment without ultrasound, US indicates ultrasonic treatment.

The crystal violet-stained biofilm of the clinical strain was uniform and compact under optical microscope. Ultrasonic treatment induced the formation of large number of craters on the biofilm surface. However, the effect of UTMD treatment was more significant; in addition to the formation of craters, peeling of large areas appears on the surface of the treated biofilm. Combined treatment of ultrasound plus vancomycin or UTMD plus vancomycin produced similar effect on biofilm morphology compared to those obtained with ultrasonic or UTMD treatment, respectively. Treatment with vancomycin or vancomycin plus microbubbles did not affect biofilm morphology (Fig. 1).

**Figure 2:**
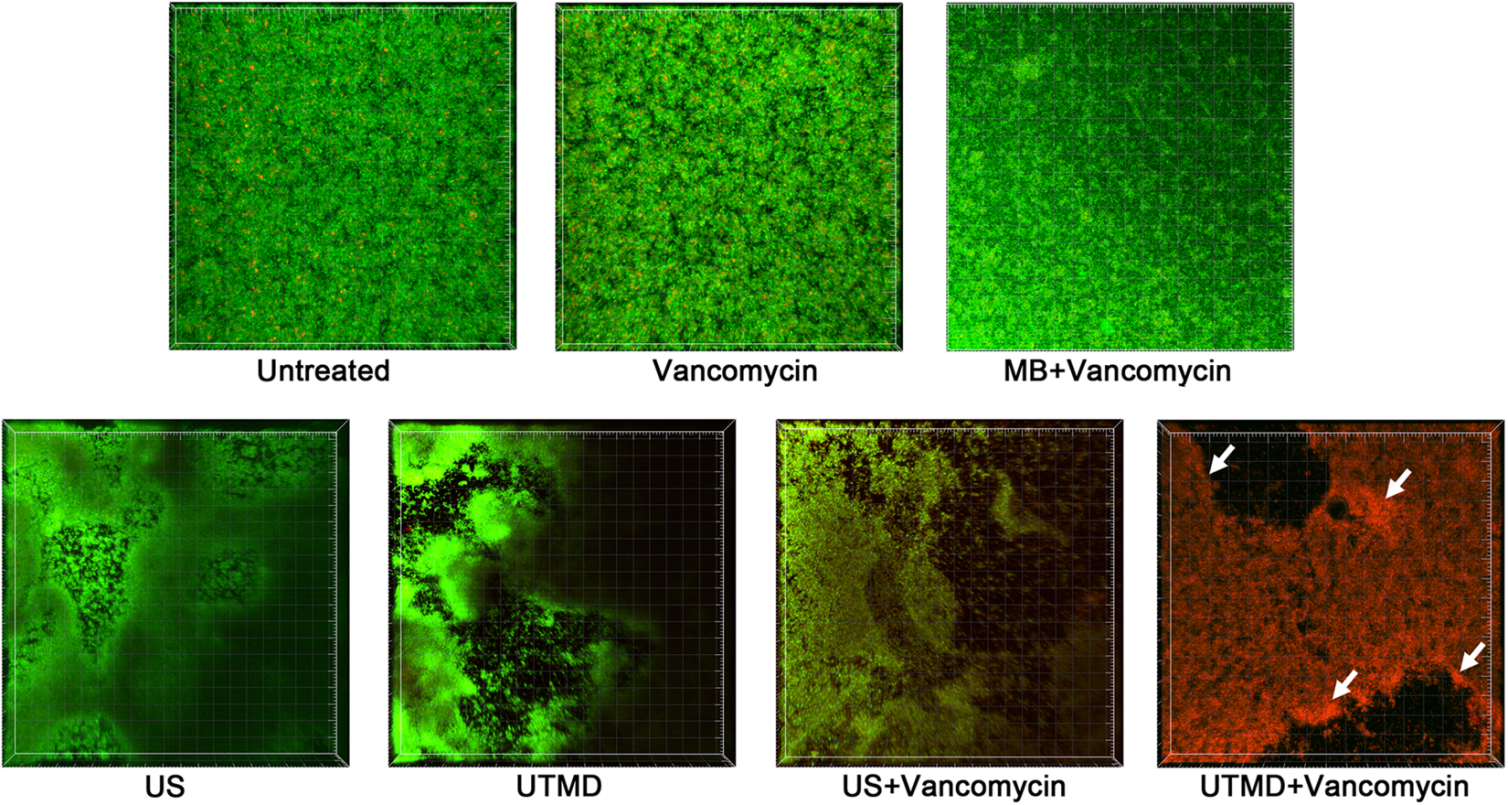
Three-dimensional structure of biofilms stained by live/dead viability staining kit. Green fluorescence indicates viable cells, whereas red fluorescence indicates dead cells. The images were three-dimensionally reconstructed using the Imaris software (Bitplane, http://www.bitplane.com) based on CLSM data at approximately 0.5 μm increments. MB: microbubble treatment without ultrasound, US: ultrasonic treatment. White arrows indicate the more gathered dead cells around the craters.

Further, confocal laser scanning microscope (CLSM) showed that the live/dead-stained biofilm of the clinical strain was relatively flat and contained viable bacteria. The surface of the biofilm after ultrasonic or UTMD treatment was rough, and the treated biofilms contained abundant craters and local peelings. The non-peeling biofilm beyond the craters still contained viable bacteria. Combined treatment of ultrasound plus vancomycin yielded similar effect on biofilm survival compared to ultrasonic treatment alone, except for a slight increase in the number of dead bacteria. However, the combined treatment of UTMD plus vancomycin produced a dramatic effect on biofilm survival; in addition to the formation of craters and local peelings, the entire biofilm is full of dead bacteria. Additionally, the dead cells were clustered more around the craters than in other areas. The control biofilms, treated by vancomycin and vancomycin plus microbubbles, showed no morphological changes under CLSM (Fig. 2).

### Effects of ultrasound and UTMD on bacterial ultrastructure in biofilms

Above studies suggested that ultrasound and UTMD, especially UTMD, not only directly damaged the *S*. *epidermidis* biofilm, but it also assisted vancomycin in eradicating bacteria residing in the biofilm. To investigate the mechanisms underlying the synergistic bactericidal effect of vancomycin and UTMD on biofilm cells and to understand the nature of the cellular damage inflicted on bacteria in the treated biofilms, transmission electron microscope (TEM) was used to study the ultrastructural changes in the treated bacteria.

**Figure 3:**
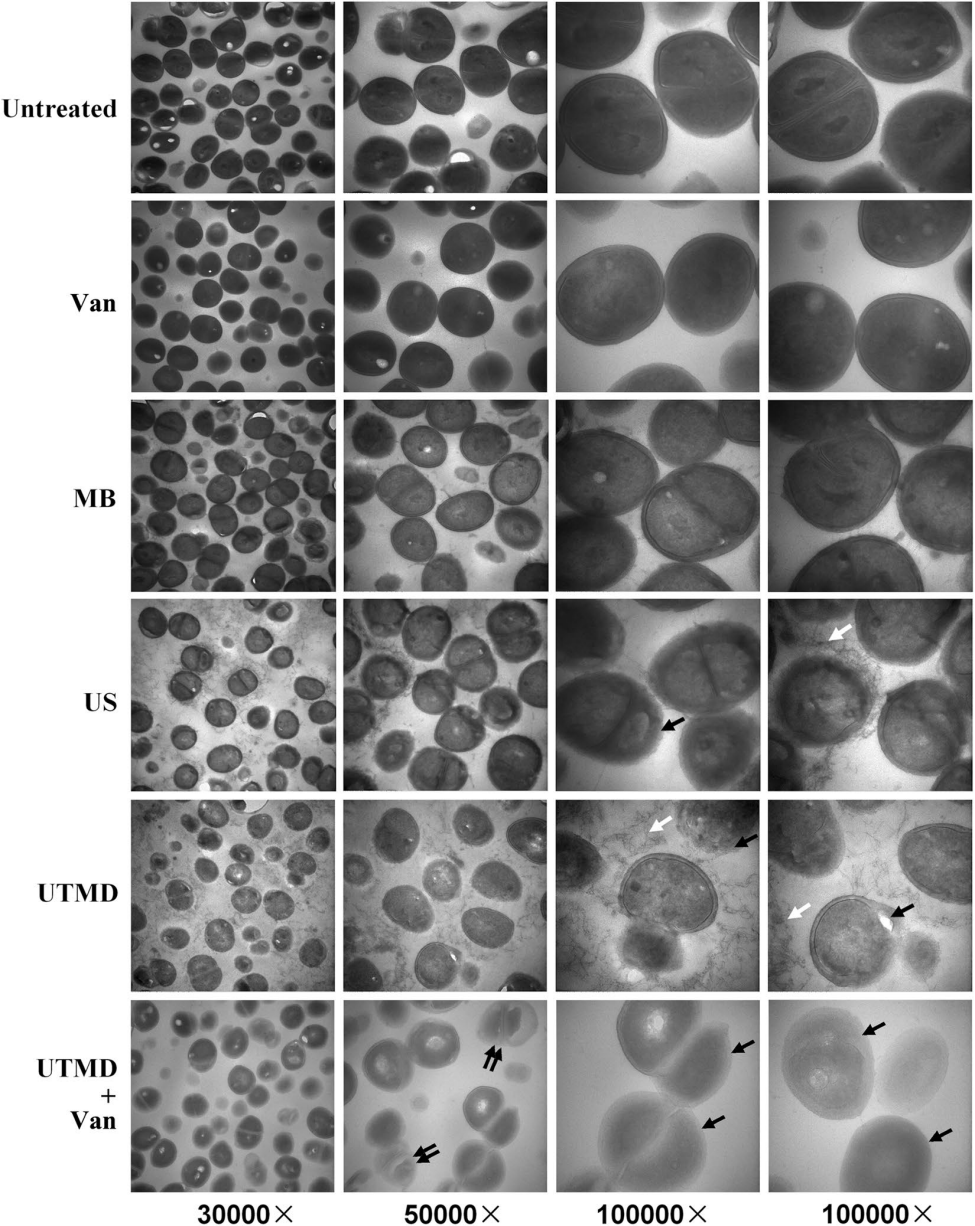
Transmission electron microscopic images of the ultrastructure of cells in the biofilm. The bacteria in the electron micrographs are magnified by 30,000× , 50,000× , and 100,000× (double views). MB: microbubble treatment without ultrasound, Van: vancomycin treatment, US: ultrasonic treatment. White arrows indicate the filaments and floccules distributed in the space between treated cells, black arrows indicate the cell wall damages, and double black arrows indicate the dissolved protoplasts.

The cellular ultrastructure of *S*. *epidermidis* in biofilm was not significantly different from those of the typical planktons. Electron micrographs showed intact cells spread on a clean background, and biofilm matrix components (extracellular polymeric substances) were not visible. However, significant cell wall damage, including local fracture and boundary blurring, were observed in several cells treated by ultrasound or UTMD, and the spaces between those treated cells were filled with filaments and floccules, indicating that the cell wall components were shed from the damaged cell wall surfaces. Further, the treatment of UTMD plus vancomycin caused most serious damages on *S*. *epidermidis* cells, the cell walls of most cells became blurred, and cell protoplasts of some cells were dissolved. Nevertheless, the space between UTMD plus vancomycin treated cells showed no filament or floccule, which was different from UTMD treated one, because these cells well further incubated for 6 hours after UTMD treatment, and the floccules might be decayed by enzymes release by cells (dead or alive). In addition, the electron density of cells after ultrasound or UTMD treatment decreased, especial after UTMD or UTMD plus vancomycin treatment. For the control groups, the ultrastructure of biofilm cells treated by microbubbles or vancomycin were similar to those of the untreated sample (Fig. 3).

### Effects of ultrasound and UTMD on metabolic activities and cell membrane integrity of *S*. *epidermidis*

The above studies revealed that ultrasound, especially UTMD, may cause direct damage to the ultrastructure of *S*. *epidermidis* cells in biofilm. To analyze this further, we used two fluorescent agents, 5-carboxyfluorescein diacetate (5-CFDA) and 5-cyano-2,3-ditolyl tetrazolium chloride (CTC), to study cell membrane integrity and changes in metabolic activity of the cells post-treatment. 5-CFDA is a cell-permeable esterase substrate that can be used both as a viability probe to measure enzymatic activity (which is required to activate its fluorescence (green)) and cell-membrane integrity (which is required for intracellular retention of their fluorescent product)^35,36^. CTC is a substrate that can be reduced into an insoluble, red fluorescent formazan product by the electron transport chain, and can be used to evaluate the respiratory activity of bacterial populations^37,38^. Increased fluorescence of 5-CFDA and CTC indicate enhancement of esterase activity and respiration while the cell membrane are still intact.

**Figure 4:**
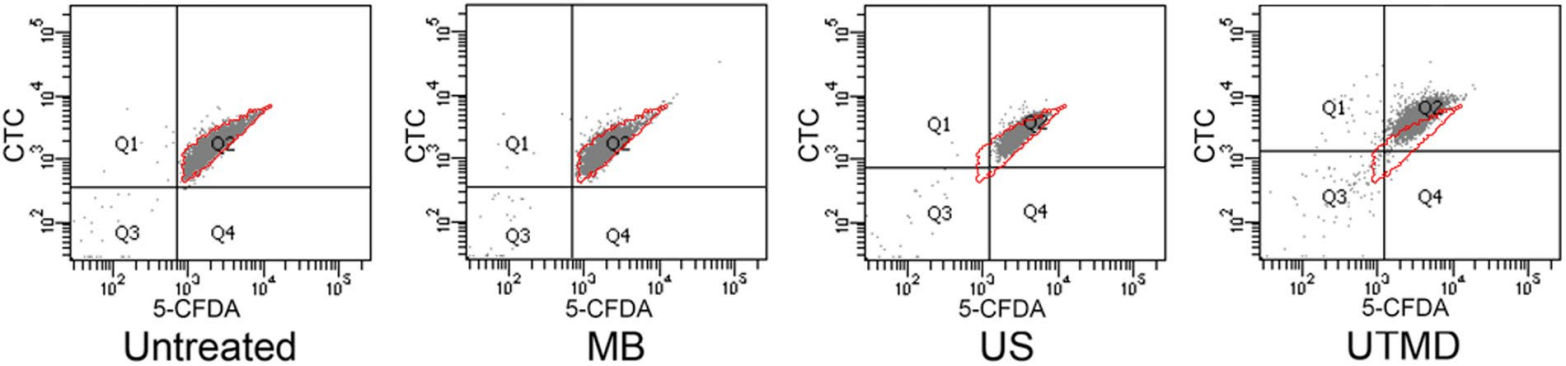
Fluorescent intensities of 5-CFDA and CTC of stained planktonic *S*. *epidermidis* detected by flow cytometry. MB: microbubble treatment without ultrasound, US: ultrasonic treatment, red borderline indicate the distribution range for the untreated cells as comparison.

First, we investigated the effects of ultrasound or UTMD on planktonic *S*. *epidermidis* before studying the possible physiological changes of the cells in the biofilm. After staining by 5-CFDA and CTC for 30 min at room temperature, fluorescence of treated planktonic bacteria were detected by flow cytometry. As shown in Fig. 4 and Table 1, either fluorescent intensities (green and red) of cells treated by ultrasound or UTMD increased than those of the untreated or microbubble- treated cells; the UTMD-treated cells showed the highest mean fluorescence intensities. This indicated that the metabolic activities of the cells damaged by ultrasound, especially UTMD, increased, while the integrity of the cell membranes was unaltered.

**Table 1:** Mean fluorescent intensities (MFI) of the Q2 population of cells showed in Fig. 4.

	5-CFDA MFI	Ratio	CTC MFI	Ratio
Untreated	2493	1	1697	1
MB	2336	0.94	1558	0.92
US	3457	1.39	2572	1.52
UTMD	4043	1.62	4610	2.72

Ratio: MFI ratio to untreated cells.

“MB”: microbubble treatment without ultrasound.

“US”: ultrasonic treatment.

**Figure 5:**
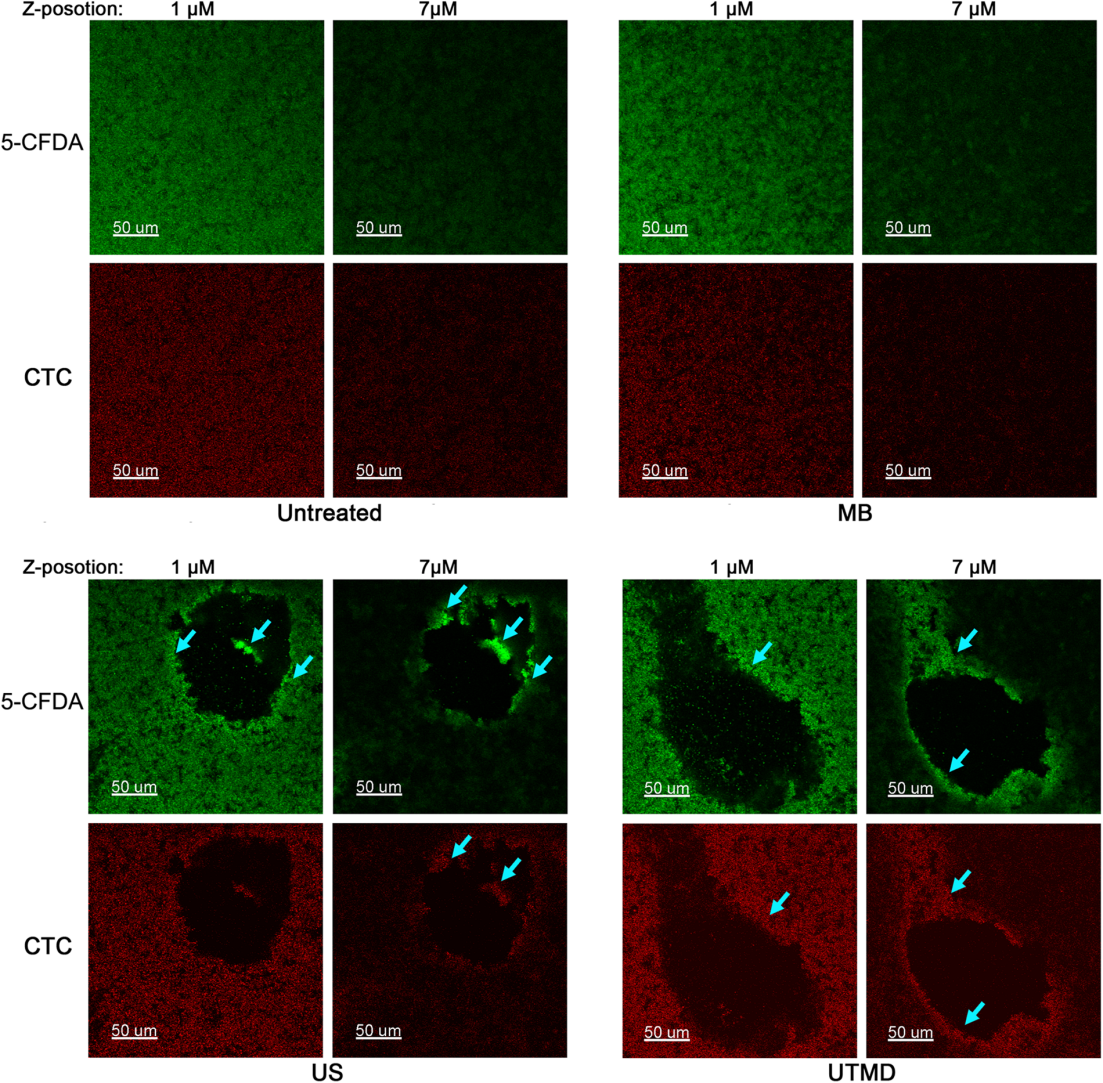
Confocal photo sets of 5-CFDA and CTC-labeled biofilms. Confocal microscopy Z-series of the biofilm were acquired in 0.5 μm increments. Photo sets with Z-position at 1 μm shows the layer located 1 μm from the bottom of the biofilm, and that at 7 μm shows the layer located near the surface of the biofilm, which is 7μm from the bottom. MB: microbubble treatment without ultrasound, US: ultrasonic treatment. Bright blue arrows indicate the further enhanced fluorescence of cells around the craters and cells that have not yet been shed in the center of the craters.

**Figure 6:**
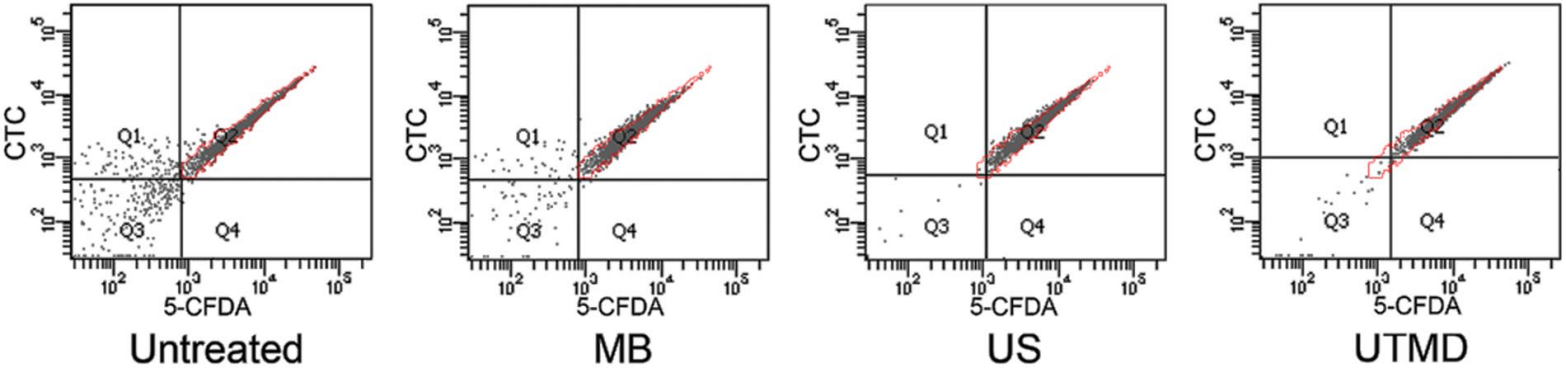
Fluorescent intensities of 5-CFDA and CTC-stained cells in scraped biofilms detected by flow cytometry. MB: microbubble treatment without ultrasound, US: ultrasonic treatment, red borderline indicate the distribution range for the untreated cells as comparison.

We further observed the 5-CFDA and CTC-labeled biofilms under a CLSM to confirm that the behavior of cells in treated biofilms were similar to those of the planktons. Unlike the planktons, only UTMD-treated biofilm showed brighter fluorescence (Fig. 5), whereas the cells in ultrasound-treated biofilm showed similar fluorescence intensities as that of the untreated sample. Further, flow cytometric analysis of fluorescent intensity of cells from manually scraped biofilm components also indicated that the increase in fluorescence occurred only for the UTMD-treated biofilm (Fig. 6 and Table 2). Furthermore, we found that the fluorescence intensity distribution for the ultrasound or UTMD-treated biofilm was different from that of the untreated control. *S*. *epidermidis* cells in the untreated biofilm or microbubble-treated control were homogeneously labeled with both fluorescent dyes, although it appeared that the fluorescence on the biofilm bottom was brighter than that on the surface as more cells aggregated at the bottom of the biofilm. However, for the ultrasound or UTMD treated biofilms, the fluorescence intensities of the cells surrounding the craters and those of cells that had not yet been shed in the center of the craters of the treated biofilms were higher (Fig. 5), demonstrating that the metabolic activities of these cells were further enhanced than those of cells in other areas. Additionally, the integrity of their cell membranes was retained.

**Table 2:** Mean fluorescent intensities (MFI) of the Q2 population of cells showed in Fig. 6.

	5-CFDA MFI	Ratio	CTC MFI	Ratio
Untreated	6822	1	3831	1
MB	5156	0.76	3088	0.81
US	7200	1.05	4064	1.06
UTMD	12346	1.81	6819	1.78

Ratio: MFI ratio to untreated cells.

“MB”: microbubble treatment without ultrasound.

“US”: ultrasonic treatment.

## Discussion

Biofilms lead to the development of antibiotic-resistant infections in medical implants and prosthetics, which renders the treatment of such infections challenging. Although the detailed mechanism underlying the drug resistance is still unknown, several studies have attempted to address this question. One group showed that the biofilm matrix protects the cells embedded in it^9^; on the contrary, other groups demonstrated that the cells embedded in the biofilm are in a dormant state^10,39,40^, and since the targets of many antibiotics are involved in metabolism-intensive processes such as cell wall synthesis, protein synthesis, replication or transcription, the dormant biofilm bacteria respond poorly to the antimicrobial effect of antibiotics.

Studies suggest that the cavitation effect of ultrasound and UTMD is the key factor contributing to their biological effects. In eukaryotes, the cavitation effect can directly damage the cell membrane and cause non-lethal tiny pores on the surface of cells, which are visible by scanning electron microscopy^41^. Drugs or biological macromolecular substances that could not cross the cell membrane prior to ultrasound or UTMD treatment, can now enter the cells though these tiny pores, thus facilitating processes such as gene transfection^41–44^. For bacteria, cavitation effect of ultrasound damages the outer membrane in cell wall and the cell membrane, and the increased permeability eases the entry of antibiotics into the periplasm and cytoplasm, which subsequently promotes bacterial cell death^45–51^. In addition, cavitation effect also increases the quantity of intracellular reactive oxygen species in bacteria that enhances the damage to bacterial cells, thereby facilitating the effect of antibiotics^52^. Several studies have attempted to explain the ultrasound or UTMD assisted- destruction of biofilms by antibiotics. The most plausible explanation is that the ultrasound damages the large molecules of the extracellular polymeric substances in the biofilm, which eases antibiotic penetration into the biofilm^31–34^. Other studies showed that UTMD enhances the activity of β-defensin 3, which inhibits the expression of biofilm-associated *icaAD* and the methicillin-resistance gene *mecA* by promoting the expression of *icaR*, thereby synergizing the lethal effect of antibiotics on biofilms *in vivo* ^53^. However, studies regarding the biological effects of ultrasound on biofilm-embedded cells are limited, and whether ultrasound changes the physiological state or transcriptional status of the bacteria prior to reducing antibiotic resistance is still unknown.

Previously, we showed that UTMD considerably assisted vancomycin in killing *S*. *epidermidis* RP62A in biofilms compared to signal ultrasound^30^. In the current study, we identified a clinical strain of *S*. *epidermidis*, and observed that its biofilm was more sensitive to UTMD plus vancomycin treatment than that of RP62A. This provides a better opportunity for studying the effects of ultrasound on bacterial biofilm as UTMD affects this clinical strain more than RP62A isolate. We found that both UTMD and ultrasound directly damage the ultrastructure of the bacterial cells in the biofilm of this strain. Obvious cell wall damage was detected on a large number of treated cells. In addition, the filaments and floccules appearing on the spaces around the treated cells could be the components shed from the damaged cell walls. Furthermore, the bacteria in the UTMD-treated biofilm showed an obvious decrease in electron density of the protoplast, unlike the cells treated with signal ultrasound. This indicated that both the cell wall and protoplasts of the bacterial cells in the UTMD-treated biofilm were severely damaged. Electron microscopy studies on *Escherichia coli* and *Staphylococcus aureus* treated with ultrasound waves also showed signs of damage on cell surface structures^45,54^, which is in agreement with our results.

Further studies on the physiological state of ultrasound or UTMD-treated bacteria revealed that the cell wall damaged bacteria still maintained their cell membranes intact after the treatment. Therefore, we concluded that the energy of the ultrasound waves or UTMD used in our experiments has no direct lethal effect on the bacteria, but they enhanced the bactericidal capacity of vancomycin, which is an antibiotic used for inhibiting bacterial cell wall synthesis. Inhibition of cell wall synthesis in cell wall-damaged cells finally caused cell death. In contrast, the flexibility of the cell membrane, unlike the rigidity of the cell wall, might be responsible for the lack of any damage to the cell membrane.

Our studies on bacterial metabolism further showed that both the biofilm-embedded and planktonic cells of this UTMD-treated clinical isolate possessed increased metabolic activities. The dormant bacteria in the biofilm were forced to recover and initiate the stress system for reparation, including enhanced metabolic activities, for repairing the injured cell walls and protoplasts. These procedures increase the sensitivity of the bacteria to vancomycin and finally cause cell death. The following observations substantiate this hypothesis: the metabolic activities of the cells embedded in biofilm were less enhanced by signal ultrasound, where less dead cells were observed in the ultrasound plus vancomycin-treated biofilm. In contrast, metabolic activities of the cells adjacent to the craters of treated biofilm were further enhanced, and dead cells clustered more around the craters than in other areas of the biofilm subjected to UTMD plus vancomycin treatment. In agreement with this result, the UTMD plus vancomycin-treated biofilm of *S*. *epidermidis* RP62A reported in our previous study also showed more dead cells in and around the craters, whereas a large number of viable cells were present in areas distant from the craters^30^. In addition, recent studies also reported similar enhancement in physiological behavior of ultrasound-treated bacteria, including increase in growth rate^55^, enhanced oxygen consumption or CO_2_ production^44,55,56^, and activation of the DNA and protein repair systems^44^.

In summary, the direct damage of bacterial cells, as well as the increase in metabolic activities, enhanced the bactericidal capacity of antibiotics to biofilm under UTMD treatment. However, in agreement with the opinion expressed in two recent reviews^15,16^, the physiological state of the activated bacteria in treated biofilms could be a double-edged sword; on one hand, it increases the sensitivity of the bacteria to antibiotics, whereas on the other hand it may assist in the dissemination of infection, which could lead to serious adverse side effects of the treatment, especially when used in association with a clinically incorrect (non-sensitive) antibiotic. Therefore, the UTMD-facilitated antibiotic treatment is still in its infancy, and requires extensive investigation before clinical implementation.

## Methods

### Bacterial strains, media, and antibiotics

The bacterial strain used in this study was a clinical isolate of *S*. *epidermidis*, which was isolated from a central venous catheter of a patient in the First Affiliated Hospital of Xi’an Jiaotong University, Xi’an, China. The drug sensitivity profile of the clinical isolate was determined using the VITEK 2 compact automatic bacterial identification and drug sensitivity analysis system (Biomerieux, Lyon, France). Tryptone soy broth medium (TSB, Oxoid, Basingstoke, United Kingdom) containing 2.5% glucose was used for culturing planktonic and biofilm *S*. *epidermidis*. Vancomycin (Sigma-Aldrich, Missouri, USA) used in the study was dissolved in distilled water after filtration through a 0.22 μm sterile filter (Millipore, California, USA) and stored in −20 °C.

### Biofilm detection of *S*. *epidermidis*

Biofilm formation of *S*. *epidermidis* was detected by *in vitro* experiments. Briefly, an overnight culture of *S*. *epidermidis* grown in TSB medium was diluted 1:200 in fresh TSB and statically incubated on polystyrene cell culture plates (Corning, New York, USA) at 37 °C overnight. After incubation, the wells were washed with PBS, fixed with methanol, and stained with 2% (w/v) crystal violet.

For microscopic observation of biofilm formation, *S*. *epidermidis* were further cultivated in FluoroDishes (FD35–100, WPI, Florida, USA), stained with a live/dead kit (containing 1 μmol/L SYTO9 and 1 μmol/L PI in normal saline, Life Technologies, Massachusetts, USA) or 5-CFDA (2 μmol/L in normal saline, Life Technologies) and CTC (1 mmol/L in normal saline, Life Technologies) before observation under a CLSM (TCS SP5, Leica, Wetzlar, Germany).

For ultrastructural observation of *S*. *epidermidis* in biofilms, cells were completely scraped off from the biofilm, collected by centrifugation, washed with PBS, and fixed using osmic anhydride. After making ultrathin sections, the ultrastructure of cells stained by uranyl acetate and lead citrate was observed under a TEM (H-7650, Hitachi, Tokyo, Japan).

### Ultrasonic treatment and ultrasound-targeted microbubble destruction

The ultrasonic instrument used in this study is an ultrasonic therapeutic device for physiotherapy (US13, Cosmogamma, Bologna, Italy). The diameter of the ultrasonic transducer is 1.25 cm, and its ultrasonic frequency is 1 MHz. The ultrasonic intensity in the experiment was set to 1 W/cm^2^, and the duty cycle was 50%. The ultrasound was transmitted though the bottom of the culture wells via coupling gel, and the treatment lasted for 10 min.

The SonoVue ultrasound microbubble contrast agent (Bracco, Milan, Italy) was reconstituted in 5 mL normal saline according to the manufacturer’s instructions. The reconstituted solution contained 2 × 10^8^–5 × 10^8^ microbubbles/mL, and the average diameter of the microbubbles was approximately 2.5 μm. The final concentration of the microbubble applied in the biofilm treatment was 30% (v/v) diluted in TSB medium, and the ultrasonic treatment conditions were the same as above.

Additionally, in the combined treatment of ultrasound plus vancomycin or UTMD plus vancomycin, the antibiotic was added before the ultrasonic or UTMD treatment, and the treated biofilms were further cultured for 6 h before observation. The final concentration of vancomycin applied to the biofilm was 100 μg/mL (25 μg vancomycin per mg biofilm mass approximately).

### Flow cytometric analysis of *S*. *epidermidis* and its biofilm components

Overnight bacterial culture was centrifuged to collect the planktonic *S*. *epidermidis*. After washing thrice with PBS, the collected bacteria were fully suspended and diluted to 0.5 McIntosh turbidity unit in fluorescent staining solution containing 0.67 μmol/L 5-CFDA and 0.33 mmol/L CTC (in normal saline) before flow cytometric analysis (Cantoll, Becton Dickinson and Company, New Jersey, USA).

For obtaining biofilm cells, overnight cultured biofilm was washed with PBS for removal of non-adherent bacteria. After ultrasonic or UTMD treatment, the biofilm was fully scraped and cells were collected after centrifugation. After washing thrice with PBS, the collected biofilm components were fully suspended and diluted to 0.5 McIntosh turbidity unit in fluorescent staining solution containing 5-CFDA and CTC before flow cytometric analysis.

### Data availability

All data generated or analysed during this study are included in this published article.
